# Evaluation of Anti-Nociceptive and Anti-Inflammatory Activities of a Heterofucan from *Dictyota menstrualis*

**DOI:** 10.3390/md11082722

**Published:** 2013-08-02

**Authors:** Ivan Rui Lopes Albuquerque, Sara Lima Cordeiro, Dayanne Lopes Gomes, Juliana Luporini Dreyfuss, Luciana Guimarães Alves Filgueira, Edda Lisboa Leite, Helena Bonciani Nader, Hugo Alexandre Oliveira Rocha

**Affiliations:** 1 Laboratory of Biotechnology of Natural Polymers (BIOPOL), Department of Biochemistry, Federal University of Rio Grande do Norte (UFRN), Natal-RN 59078-970, Brazil; E-Mails: ivanruilopes@gmail.com (I.R.L.A.); sara-cordeiro@hotmail.com (S.L.C.); dayanne_gomes@hotmail.com (D.L.G.); lucianagalves@hotmail.com (L.G.A.F.); eddaleite@cb.ufrn.br (E.L.L.); 2 Graduate Program in Health Sciences, Federal University of Rio Grande do Norte (UFRN), Natal-RN 59078-970, Brazil; 3 Department of Biochemistry, Federal University of São Paulo (UNIFESP), São Paulo-SP 04044-020, Brazil; E-Mails: jdreyfuss@gmail.com (J.L.D.); hbnader.bioq@epm.br (H.B.N.)

**Keywords:** fucan, brown seaweed, pain, analgesic, dictyotales

## Abstract

Fucan is a term that defines a family of homo- and hetero-polysaccharides containing sulfated l-fucose in its structure. In this work, a heterofucan (F2.0v) from the seaweed, *Dictyota menstrualis*, was evaluated as an antinociceptive and anti-inflammatory agent. F2.0v (20.0 mg/kg) inhibits 100% of leukocyte migration into the peritoneal cavity after chemical stimulation. However, F2.0v does not alter the expression of interleukin-1 beta (IL-1β) and interleukin-6 (IL-6), as well as tumor necrosis factor alpha (TNF-α). F2.0v (20.0 mg/kg) has peripheral antinociceptive activity with potency similar to dipyrone. On the other hand, it had no effect on pain response on the hot plate test. Confocal microscopy analysis and flow cytometry showed that F2.0v binds to the surface of leucocytes, which leads us to suggest that the mechanism of action of anti-inflammatory and antinociceptive F2.0v is related to its ability to inhibit the migration of leukocytes to the site of tissue injury. In summary, the data show that F2.0v compound has great potential as an antinociceptive and anti-inflammatory, and future studies will be performed to further characterize the mechanism of action of F2.0v.

## 1. Introduction

The main function of the sensory system is to keep the brain informed of external conditions, the environment and internal conditions of the body. Among the sensations of the body, pain can be classified as one of the most important for the maintenance of homeostasis. The feeling of pain serves as an alarm, which leads, in most cases, to an affected individual having an avoidance behavior, especially when it is sharp and intense, which reduces the damage to the attacked area of the body [1]. 

The term, nociception, being related to the recognition of pain signals through the nervous system, is complexity and is a result of evolutionary pressure, experienced by species, to prevent damage to organisms. The process of nociception and pain involves diverse mechanisms, and it is obvious that a single drug will not be able to relieve the pain completely. An effective plan includes different classes of analgesic drugs acting at different points of the pathophysiological mechanisms that involve pain. Although the pain has its protective effect, the persistent soreness can lead to negative behaviors, such as depression and irritability, causing social and economic problems [1]. 

Although there are several drugs currently being used as modulators of the nociceptive system, there is always a search for new more potent and safe drugs, which may be used in specific situations. Furthermore, in this search, a tool that has performed with great value for the identification of compounds is anti-nociceptive animal models [1]. 

Natural products are sources of diverse bioactive molecules. In this context, one can mention the seaweeds. Furthermore, among the bioactive compounds synthesized in greater amounts by seaweeds, the sulfated polysaccharides stand out. They are located in the mucilaginous matrix of seaweeds, and their biological function is related to the solar protection against dehydration during periods of low tide; they provide greater flexibility to the seaweeds, like its growth in the aquatic environment and sufficient rigidity to remains extended and, thus, capturing light and nutrients more effectively [2]. Among the sulfated polysaccharides from seaweed, those that stand out, because they have pharmacological activity, are fucans. They are a family of homo- and hetero-polysaccharides having in its constitution monomer sulfated l-fucose [3]. Recent studies have demonstrated that fucans presented various pharmacological activities, such as: anticoagulant [4], antioxidant [5], immune modulator [6], anti-viral [7,8], anti-inflammatory [9] and anti-tumoral [10].

*Spatoglossum schröederi* is a seaweed from the *Dictyotaceae* order and synthesizes three different types of sulfated fucans, which were named fucans A, B and C, according to their electrophoretic mobility in agarose gel in 1,3-diaminopropane acetate buffer [11]. These fucans exhibit antiproliferative [12], anti-adhesive [13] and antithrombotic activities [14]. Recently, Farias and colleagues [15] demonstrated that one of the fucans from *S. schröederi* presents anti-nociceptive activity. At best, we know this is a unique study that analyzed the anti-nociceptive activity of fucans. *Dictyota menstrualis* is a different seaweed from the *Dictyotaceae* order; it is found in almost all of the Brazilian coast (about 8000 km), as well as in the Caribbean and Mexico. This, added to its constant presence and biomass production, makes this organism an excellent choice for prospecting bioactive compounds. Studies with fucans-rich extract from this seaweed showed that it has antioxidant activity *in vitro* and cytotoxicity against tumor cells (HeLa) [16].

In a previous work, we extracted and purified heterofucans from *D. menstrualis* [17]. Those that were obtained in larger quantities were named fucans F1.0v, F1.5v and F2.0v. The fucans, F1.0v and F1.5v, have presented anticoagulant activity. However, the fucan known as F2.0v showed no anticoagulant activity, which allows the use of this fucan for other new applications, since the anticoagulant effect could be an unwanted side effect. Thus, this study was aimed at evaluating the potential anti-inflammatory and antinociceptive activities of fucan F2.0v from the seaweed, *Dictyota menstrualis.*

## 2. Results and Discussion

### 2.1. Obtaining Fucan F2.0v from *D. menstrualis*

**Figure 1:**
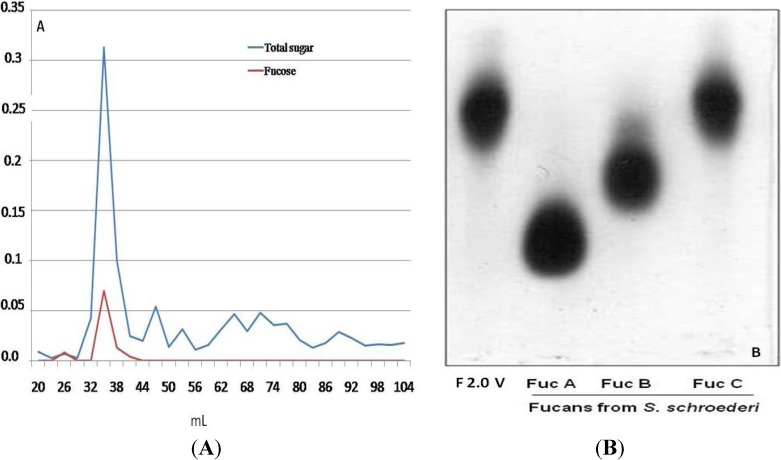
(**A**) Elution profile of F2.0v in gel filtration chromatography (A). Gel filtration chromatography fractions of a 1 mL/tube were collected and checked by the method of phenol/sulfuric [18], as well as by the presence of fucose [19]; (**B**) F2.0v representative agarose gel electrophoresis in 1,3-diaminopropane acetate buffer, stained with toluidine blue.

The polysaccharides of *D. menstrualis* were extracted by proteolysis at 60 °C followed by acetone fractionation and ion exchange chromatography. The fucan, F2.0v, was precipitated with two volumes of acetone, as described in the Methods. Thereafter, it was subjected to ion exchange chromatography on diethylaminoethanol cellulose (DEAE-cellulose) and eluted with increasing concentrations of NaCl (0.3–4.0 M). The elution profile was monitored by measurement of total sugars [18] and fucose [19]. Only using 3.0 M of NaCl was a peak of sugar observed. This material was dialyzed, lyophilized and subjected to gel filtration chromatography on a Sephadex G-75. The elution profile can be seen in Figure 1A; again, only one peak was identified. This fraction was dialyzed, dried, suspended in distilled water and subjected to agarose gel electrophoresis (see Methods). As can be seen in Figure 1B, fucan 2.0v is shown as a single band with electrophoretic mobility similar to the seaweed fucan C (Fuc C), from *Spatoglossum schröederi*, which confirms the homogeneity of F2.0v and its identity as fucan C. The amount of F2.0v obtained after purification steps correspond to 0.1% of seaweed dry weight.

Chemical analysis showed that F2.0v is composed by fucose:xylose:galactose:sulfate in the ratio of 1:0.4:1.5:1.3, respectively; traces of glucuronic acid have also been identified. The presence of protein in the sample was observed. This composition is very similar to Fuc C from *S. schröederi* [20], and in both cases, the two fucans are sulfated galactofucans. Such polymers are not as common; however, there is a description of galactofucans in seaweeds from the Laminariales order (*Undaria pinnatifida* [7], *Saccharina longicruris* [21]), the Ectocarpales order (*Adenocystis utricularis* [8]), the Dictyotales order [13,20] and the Fucales order (*Sargassum horneri* [22]). Furthermore, antithrombotic [18] and anti-viral [7] activities have been described for these heterofucans.

### 2.2. Infrared Analysis of F2.0v

**Figure 2:**
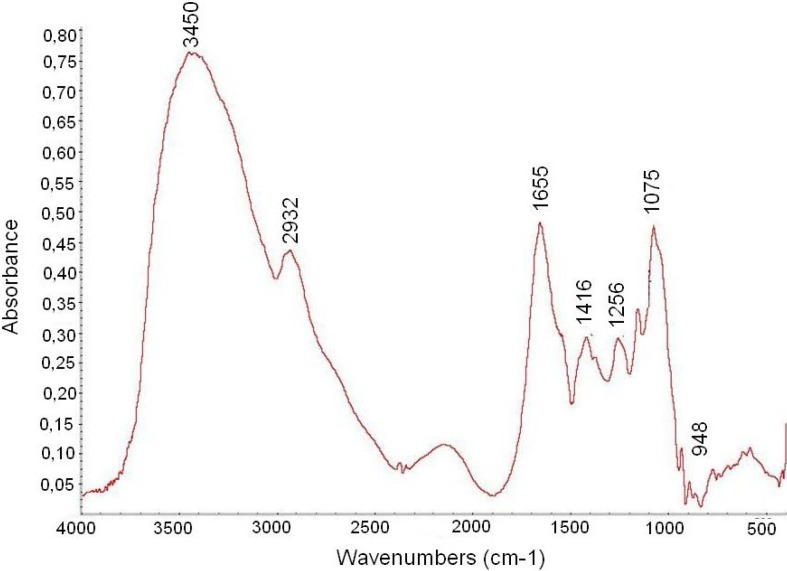
FTIR spectra of F2.0v from *D. menstrualis*.

The FTIR analysis of F2.0v is shown in Figure 2. Characteristic sulfate absorptions were identified in the FTIR spectra of compounds: Bands around 1256 cm^−1^ for asymmetric S=O stretching vibration and bands around 1075 cm^−1^ for symmetric C–O vibration associated with a C–O–SO_3_ group. The peaks at 810–850 were caused by the bending vibration of C–O–S [23]. At 3000–3400 cm^−1^, Fuc C showed bands from the stretching vibration of O–H and C–H, respectively [24], at 2932 cm^−1^ and Fuc C showed stretching vibrations of CH_2_ [25]. The peak of the C–H symmetric deformation vibration was at 1416 cm^−1^ [26]. A band at 1655 cm^−1^ was assigned to the antisymmetric stretching vibration of the COO– of glucuronic acid [3], which is overlapped with the vibration of water. 

### 2.3. Anticoagulant Activity

Fucans from various seaweeds, including different *Dictyotales* [3,4], possess anticoagulant activity. In this way, we evaluated the anticoagulant activity of the Fuc C test by Prothrombin time (PT) and Partial thromboplastin time (APTT). However, in all conditions evaluated (10 to 100 μg/mL), F2.0v showed no anticoagulant activity (data not shown).

### 2.4. Anti-Inflammatory Activity

#### 2.4.1. Fuc C Inhibits Leukocyte Migration into the Peritoneal Cavity

Heparin, a sulfated polysaccharide, has great anti-inflammatory potential. However, heparin is not used in the clinic as an anti-inflammatory drug, due to its potent anticoagulant activity, which could result in hemorrhagic complications. It has been proposed that sulfated polysaccharides similar to heparin with no anticoagulant activity could be used as an anti-inflammatory agent [27]. Moreover, several fucans have been described as anti-inflammatory agents, but like heparin, these polysaccharides possess anticoagulant activity [6].

**Figure 3:**
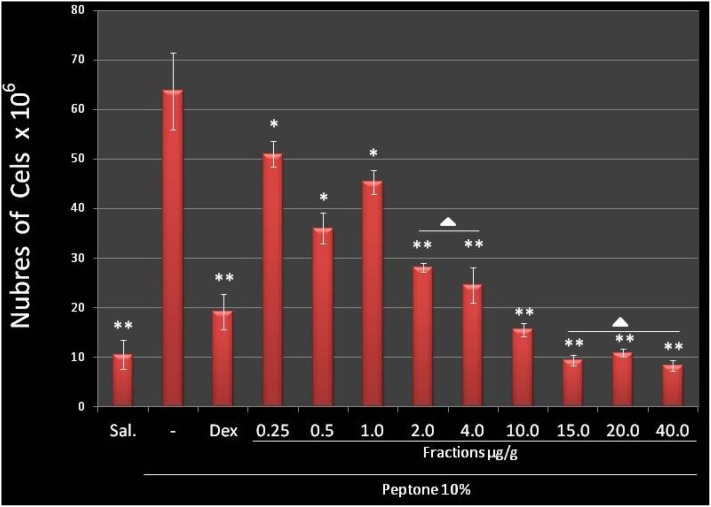
The effect of F2.0v in the migration of leukocytes in mice with induced peritonitis peptone. The bars show the media of the number of leukocytes per mL of peritoneal fluid, 6 h after intra-peritoneal administration of peptone. Peptone (10%) and dexamethasone (1.0 μg/g) are the negative and positive control, respectively. The effect of F2.0v when compared to the positive control (* *p* < 0.05; ** *p* < 0.001). ∆: There are no significant differences when compared to each other.

Since F2.0v from *D. menstrualis* presented absent anticoagulant activity, we decided to evaluate its anti-inflammatory potential. According to Lima and colleagues [28], an excellent indicator of anti-inflammatory activity of new compounds is the peritoneal cell migration inhibition in acute inflammation models. In the present study, it was verified that F2.0v polysaccharide is be able to inhibit the migration of leukocytes into the mice peritoneal cavity after stimulation with peptone in all tested concentrations (Figure 3). After 15 mg/kg, the inhibition reached a plateau, since concentrations of 15, 20 and 40 mg/kg showed no significant differences when compared to each other; compared to the saline group, 15, 20 and 40 mg/kg showed no substantial differences, pointing out that F2.0v, in these concentrations led to 100% of inhibition of leukocyte migration induced by peptone. In addition, F2.0v at 15 mg/kg was more potent than the positive control.

#### 2.4.2. F2.0v Does Not Influence the Production of Pro-Inflammatory Cytokines

Many compounds inhibit leukocyte migration into the abdominal cavity by the inhibition of pro-inflammatory cytokines, which act as chemoattractants. Many fucans have the property of inhibiting the synthesis of cytokines, such as a fucan extracted from *Ascophyllum nodosum*, which has the ability to modulate the production of TNF-α and IL-6 when stimulated with lipopolysaccharides (LPS) [29]. Therefore, in order to understand the mechanism of leukocyte migration evoked by F2.0v, we evaluated the effect of this fucan in cytokine (TNF-α, interleukin 1β (IL-1β) and interleukin 6 (IL-6)) release. For this experiment, murine macrophages were incubated with F2.0v at different concentrations (10, 50, 100 and 200 μg/mL) for 24 h. In addition, 3-(4,5-dimethylthiazol-2-yl)-2,5-diphenyltetrazolium bromide (MTT) test showed no cytotoxicity (viability more than 96%), independently of the concentration of F2.0v (data not shown). Therefore, macrophages were exposed to F2.0v (10, 50, 100 and 200 μg/mL) for 24 h in the presence or absence of LPS. The analysis showed that F2.0v alone did not influence the release of TNF-α, IL-1β and IL-6. Furthermore, F2.0v does not interfere with the stimulatory effect of LPS (data not shown). These data show that the mechanism of action of F2.0v as an anti-migratory agent is not related to modulation of secretion of cytokines.

#### 2.4.3. Fuc C Binds to Murine Leukocyte Surface

**Figure 4:**
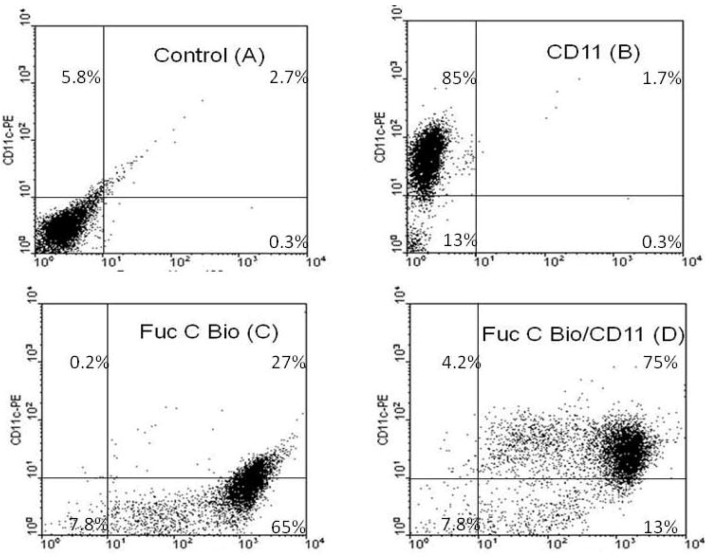
F2.0v binds leukocytes. Equal number of cells from peritoneal lavage were treated with phosphate buffered saline (PBS) (**A**), C11 antibody (**B**), biotinylated F2.0v (**C**) or both (**D**) and analyzed by flow cytometry. Similar results were obtained in three independent experiments.

The anti-migratory effect of fucans is due to the binding to the leukocyte surface, preventing the roll of these cells in the vessels’ lumen [6]. To verify the mechanism by which F2.0v exerts an effect as an anti-migratory agent, we covalently linked biotin to F2.0v and used the biotinylated F2.0v as a probe to identify the binding sites at the leukocytes surfaces. Figure 4B shows that 85% of the cells are positive for CD11, a specific marker for polymorphonuclear cells. Regarding the efficiency of biotinylation, when leukocytes were exposed to biotinylated F2.0v, a 92% (the sum of 27 and 65%; see Figure 4C) of positive cells were observed. The double labeling tests, staining both F2.0v and CD11, showed the 75% of the cells from peritoneal lavage were double positive, which indicates that the Fuc C binds itself primarily to polymorphonuclear cells. Only 4.2% of total cells from peritoneal lavage were positive only for CD11 (Figure 4D).

**Figure 5:**
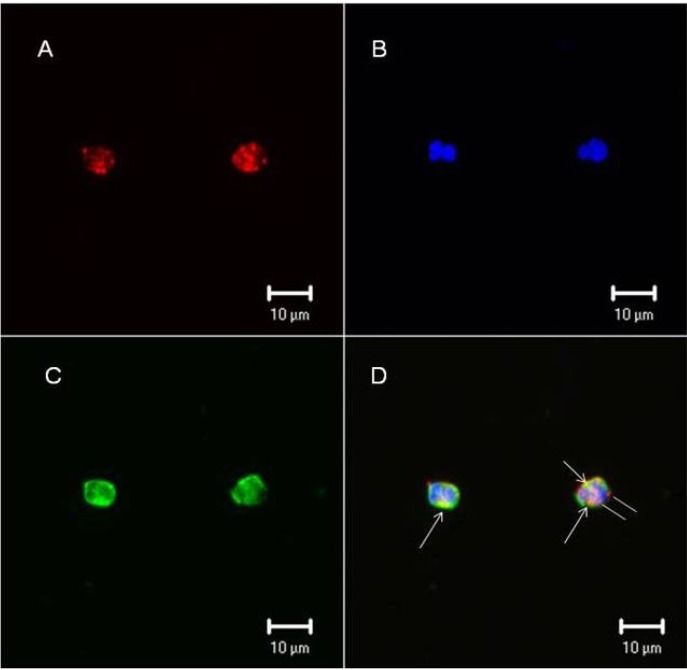
F2.0v binds leukocyte cell surface. (**A**) Biotinylated F2.0v is shown in red; (**B**) Nuclei are shown in blue, stained with DAPI; (**C**) WGA-lectin conjugated with FITC is shown in green; (**D**) Merge of the three images and the yellow color indicates colocalization of F2.0v and WGA-lectin. Similar results were obtained in two independent experiments.

Confocal microscopy tests were performed in leukocytes from human blood to confirm whether F2.0v binds the leukocyte surface. Figure 5 clearly shows that F2.0v binds the cell surface. Biotinylated F2.0v was incubated with streptavidin conjugated with Alexafluor 594, and the staining is shown in red (Figure 5). In order to locate the binding of F2.0v, a triple staining was performed by using wheat germ agglutinin (WGA) lectin conjugated with fluorescein isothiocyanate (FITC) and a nuclear staining in blue using 4′,6-diamidino-2-phenylindole (DAPI). The lectin staining is shown in green and is specific for *N*-acetyl glucosamine from the cell surface. The merge of images showed a colocalization of WGA-lectin and F2.0v, depicted by yellow dots. This result confirms that F2.0v binds to the leukocyte surface.

**Figure 6:**
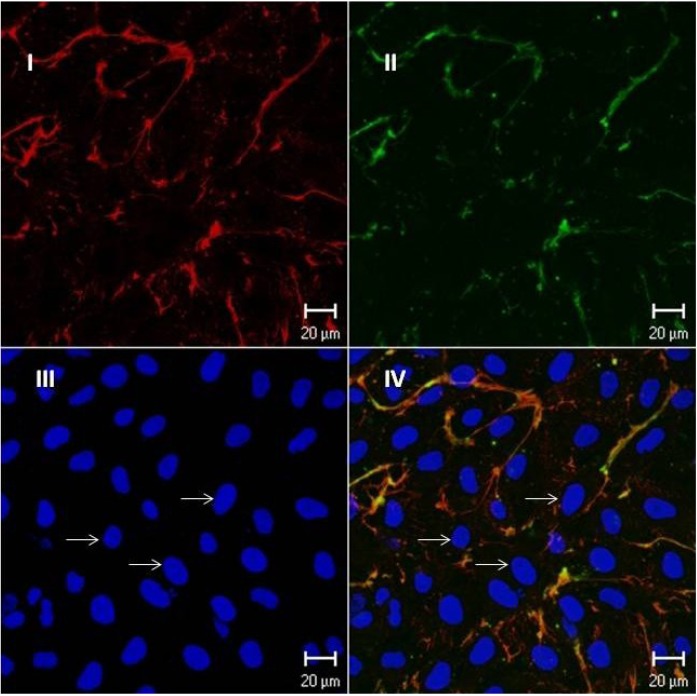
Endothelial cells exposed to biotinylated fucan; the cells were grown for three days and exposed to biotinylated fucan. The image was obtained by confocal microscopy. Barr = 20 μm. Similar results were obtained in two independent experiments. (**I**) The cells were exposed to biotinylated fucan and revealed with streptavidin conjugated with Texas Red; (**II**) Fibronectin revealed with anti-fibronectin conjugated with FTIC; (**III**) Nucleus stained in blue with DAPI; (**IV**) Superposition of images A and B. Arrows indicate the cell nucleus stained with DAPI.

In order to verify if F2.0v would be able to bind to other cells, we incubated this biotinylated polymer with endothelial cells. As seen in Figure 6, the biotinylated fucan co-localizes with fibronectin, an extracellular matrix protein. We were not able to identify the biotinylated fucan at the cell surfaces. 

**Figure 7:**
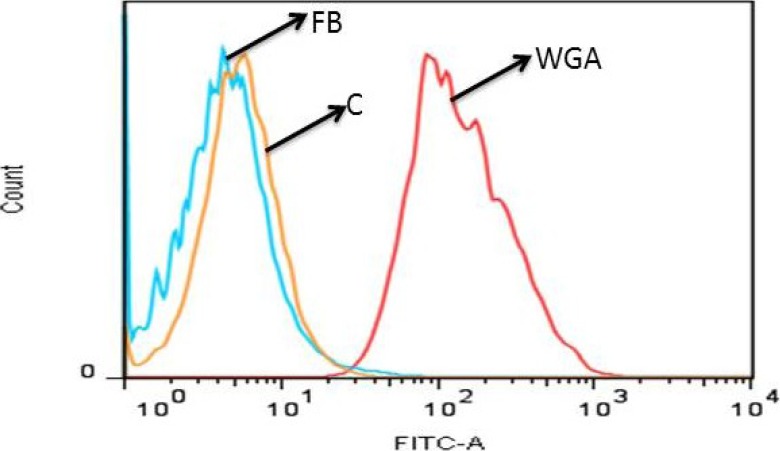
Biotinylated fucan does not bind to the endothelial cell surface; the biding of fucan on the endothelial cell surface was analyzed by flow cytometry.WGA: FITC conjugated WGA-lectin; C: endothelial cell; FB: endothelial cell exposed to biotinylated fucan. A representative out of three experiments yielding identical results is shown.

In another set of experiments, cells were removed with EDTA and assayed in suspension. Cells were incubated with biotinylated fucan B for 1 h at 4 °C and analyzed by flow cytometry, as described in the Methods. As positive control, we have used FITC conjugated WGA-lectin. Figure 7 shows, again, that biotinylated fucan does not bind to the cell surface.

### 2.5. Anti-Nociceptive Activity

Since F2.0v has an anti-inflammatory activity and the intimate relationship between the inflammatory process and the development of pain is known, the analgesic and/or anesthetic activity of F2.0v was verified. Two animal models were used to investigate pain: The acetic acid-induced writhing and hot plate tests. These tests have been reported, and they bring information from peripheral and central activity, respectively [30].

**Figure 8:**
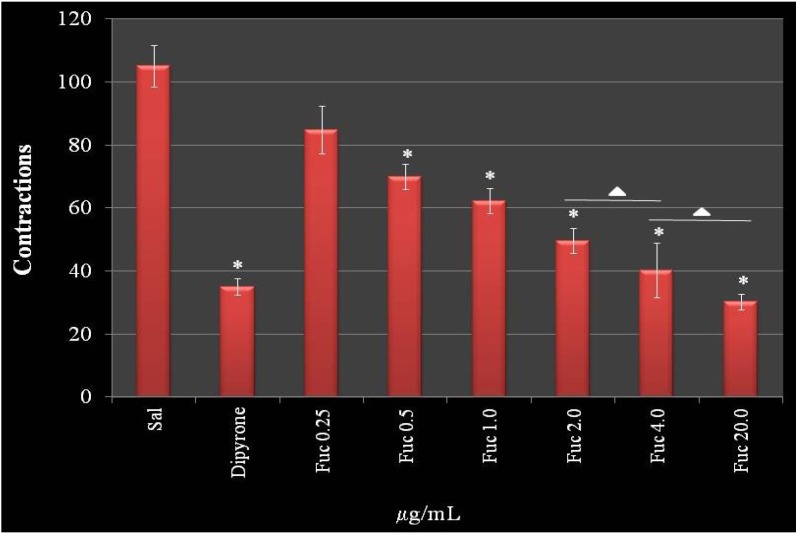
The effect of administration of F2.0v in the number of abdominal contractions in acetic acid-induced mice. Data are expressed as the mean ± S.E.M. of six animals for each group. * *p* < 0.001 indicates significant difference from the saline group. ∆: There are no significant differences when compared to each other.

Figure 8 demonstrates a dose-dependent effect of F2.0v in reducing nociceptive sensation in the chemical induction test. This effect reached the maximum of 61.2% at a concentration of 4.0 mg/kg of animal, which was similar with higher concentrations (20.0 mg/kg). In addition, there was no difference between the effect observed with 2.00 mg/kg and 4.0 mg/kg. Interestingly, at 4.0 mg/kg, the protective effect of F2.0v was similar to dipyrone (65.6%), a reference drug used in tests of peripheral analgesia. These data show that F2.0v has a peripheral antinociceptive activity.

Sulfated galactans from the seaweed, *Gracilaria cornea*, also decreased the number of contractions to around 65%, but in a higher dose (9.0 mg/kg) [30]. On the other hand, sulfated galactans from the seaweed, *Champia feldmannii*, showed a more potent effect in decreasing contractions than F2.0v, around 80%, in a lower concentration when compared to F2.0v. However, this galactan has an anticoagulant activity [31]. 

**Figure 9:**
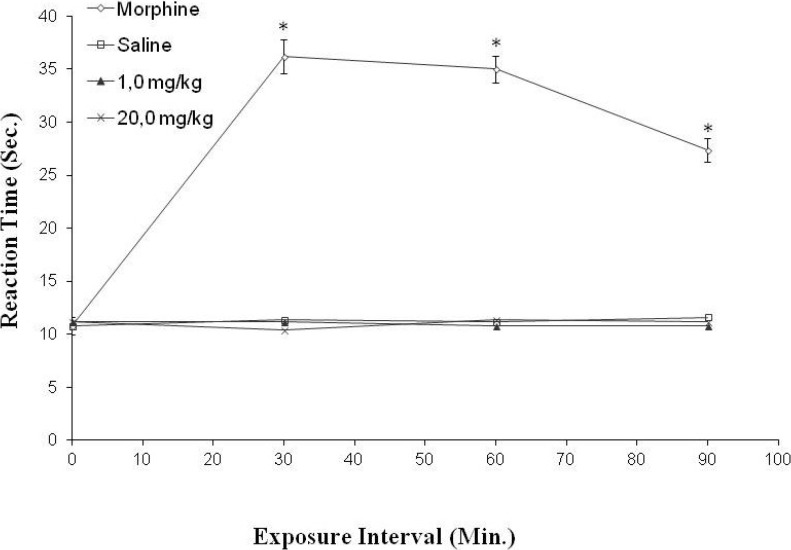
The effect of administration of F2.0v in the reaction time of the hot place test. Data are expressed as the mean ± S.E.M. of six animals for each group. * *p* < 0.001 indicates significant difference from the saline group.

The hot plate test showed that F2.0v had no effect in the tested concentrations. The results were not significantly different from the negative control. Taken together, F2.0v was not able to decrease the sensation of pain (Figure 9).

The fucan of *Spatoglossum schröederi*, the only other fucan where an analgesic effect was analyzed, also showed peripheral antinociceptive effect [15]. These data suggest that fucans act as peripheral anti-nociceptives. However, there are few data to sustain this hypothesis. 

In this study, we show that F2.0v does not diminish the sensation of pain via the central nervous system, but is an antinociceptive when the pain is peripheral. Previous studies have suggested that induction of contraction by acetic acid has an indirect mechanism of action; it induces the release of endogenous mediators that lead to stimulation of the nociceptive neurons sensitive to non-steroidal anti-inflammatory drugs and opioids, and many of these mediators are derived from immune cells that migrate to the focus of irritation, such as TNF-α and IL-1β [32]. The data obtained using cultures of macrophages have shown that F2.0v does not stimulate the synthesis of these pro-inflammatory cytokines. Moreover, F2.0v decreases the amount of migratory cells (leukocytes) to the peritoneal cavity, which, consequently, could lead to decreased amounts of endogenous mediators. Therefore, we believe that F2.0v has peripheral antinociceptive activity by decreasing the migration of leukocytes to the focus of irritation. The data of Ribeiro *et al*. [32] corroborate our hypothesis. These authors found a significant effect of 0.6% acetic acid in inducing the feeling of pain and decreased when it was promoted in a low number of leukocytes in the peritoneal cavity, suggesting that induced nociceptive activity promoted by acetic acid occurs through a mechanism dependent on the presence of leukocytes. 

## 3. Experimental Section

### 3.1. F2.0v Purifying

The brown seaweed, *Dictyota menstrualis*, was collected on Búzios beach (05°58′23″S–35°04′97″W), Rio Grande do Norte State (Brazil’s Northeast), in November 2010. Immediately after collection, algae were identified by Valquiria P. Medeiros from the Centro de Biociências/UFRN, Natal, RN, Brazil. It was brought to the laboratory for Biotechnology Natural Polymers (BIOPOL), which was clean, dried and crushed. One hundred grams of tissue were treated with dry acetone to remove lipid contaminants, after evaporation of acetone; the material was subjected to protein digestion, according to Rocha *et al*., 2005 [11], for the release of polysaccharides. They were separated by an increasing precipitation with acetone, as described in Albuquerque *et al*., 2004 [17]. 

The fraction precipitated with 2 volumes of acetone, which contained F2.0v, was solubilized in NaCl (0.25 M) and subjected to ion exchange chromatography DEAE cellulose. The material was eluted with increasing concentrations of NaCl (from 0.3 to 3.0 M). After dialysis and lyophilization, the obtained fraction with 2.0 M NaCl, which contained F2.0v, was further purified by molecular sieving in Sephadex G-75 (140 × 2.6 cm). About 200 mg of fraction, dissolved in 2 mL of water, were applied to the column and eluted with a solution of 0.2 M acetic acid and 0.15 M NaCl. Fractions of 1 mL were collected and tested by the phenol/H_2_SO_4_ reaction [18] and by detection of fucose using the Dische method [19]. Fractions containing sulfated fucose-rich polysaccharides were pooled, dialyzed against distilled water and lyophilized.

### 3.2. Agarose Gel Electrophoresis

Agarose gel electrophoresis of fucans was performed in 0.6% agarose gel (7.5 cm × 10 cm × 0.2 cm thick) prepared in 0.05 M 1,3-diaminopropane acetate buffer, pH 9.0, as described previously [33]. Aliquots of the polysaccharides (about 50 μg) were applied to the gel and subjected to electrophoresis. The gel was fixed with 0.1% cetyltrimethylammonium bromide solution for 2 h, dried and stained for 15 min with 0.1% toluidine blue in 1% acetic acid in 50% ethanol. It was then distained using the same solution without the dye.

### 3.3. Chemical Analysis and Monosaccharide Composition

Total sugars were estimated by the phenol-H_2_SO_4_ reaction [18] using l-fucose as the standard. After acid hydrolysis of polysaccharides (4 M HCl, 100 °C, 6 h), sulfate content was determined according to the gelatin-barium method [34], using sodium sulfate as standard. Protein content was measured by Spector’s method [35]. To determine the best polysaccharide acid hydrolysis using HCl, that is, where polymer degradation occurs without destroying monosaccharaides released by this degradation, F2.0v was hydrolyzed with HCl 0.5 M, 1 M, 2 M and 4 M, at 30 min, 1, 2 and 4 h, respectively. A temperature of 100 °C was maintained in all hydrolysis conditions. The material was later neutralized, dried and resuspended in water, and reducing sugars were determined, as described in Camara *et al*. [3]. The best hydrolysis condition was 2 M of HCl for 2 h. Thus, F2.0v hydrolyzed (2 M HCl, 100 °C, 2 h) and their sugar composition was determined by a LaChrom Elite^®^ HPLC system from VWR-Hitachi with a refractive index detector (RI detector model L-2490). A LichroCART^®^ 250 column (250 mm × 40 mm) packed with Lichrospher^®^ 100 NH_2_ (5 μm) was coupled to the system. The sample mass used was 0.2 mg, and the analysis time was 25 min; as references, the following sugars were analyzed: arabinose, fructose, fucose, galactose, glucose, glucosamine, glucuronic acid, mannose, mannuronic acid, rhamnose and xylose. Dexamethasone (Decadron, Aché; Campinas, SP, Brazil).

### 3.4. Fourier Transformed Infrared Spectroscopy (FTIR)

Sulfated polysaccharide (5 mg) was mixed thoroughly with dry potassium bromide. A pellet was prepared, and the infrared spectrum was measured on a Thermo Nicolet spectrometer instrument, model Nexus 470 FTIR, between 500 and 4000 cm^−1^. Thirty-two scans at a resolution of 4 cm^−1^ were averaged and referenced against air.

### 3.5. Anticoagulant Activity

APTT and PT tests were performed, as described in [36]. Unfractionated heparin (Sigma, São Paulo, Brazil) was used as the standard.

### 3.6. Animals and Cells

Male BALB/c mice, 6 to 8 weeks old, were bred and maintained in cages with food and *ad libitum* water, in the animal housing facility of the Department of Biochemistry, Federal University of Rio Grande do Norte, Natal, Brazil.

The experimental protocol for animal use was submitted to the Ethics Committee on Animal Use, approved under protocol number 003/2011 by the Federal University of Rio Grande do Norte.

The cells used for the present work were an endothelial cell line from the rabbit thoracic aorta. The cells were grown in F-12 medium (Life Technologies, Rockville, MD, USA), supplemented with 10% FBS (fetal bovine serum) (Cultilab, São Paulo, Brazil), 100 μg/mL streptomycin and 100 IU/mL penicillin (Sigma) at 37 °C in atmosphere of 2.5% CO_2_.

### 3.7. Migration of Leukocytes into the Peritoneal Cavity

The animals (mice—Balb C, weighing 20 to 25 g) were separated from the colony 12 h before the experiment for adaptation. Random groups containing 5 animals were formed. Groups were defined as substances administered to the negative control (saline i.v. and 10% peptone i.p.); positive control (dexamethasone (1.0 μg/g) i.v. and i.p.); saline group (saline i.v. and i.p.); test group (F2.0v was administered intravenously (100 μL) under different doses (from 0.25 to 40 mg/kg)). Thirty minutes later, peptone 10% (1 mL) was administered intra-peritoneally. After the migration time (6 h), the animals were euthanized with a high dosing of sedative. The cells were collected by peritoneal lavage with 10 mL of saline and counted. The result was expressed as the number of cells per mL of peritoneal lavage.

### 3.8. Quantification of Cytokines

Cells (1 × 10^6^ in duplicate) obtained from peritoneal lavage were cultured in 24-well plates at 37 °C and 5% CO_2_ in medium RPMI-1640, supplemented with fetal bovine serum (10%) containing LPS (lipopolysaccharides from the outer membrane of Gram-negative bacteria) (50 ng/mL) in the absence (positive control) or presence of Fuc C at different concentrations (0.25 to 20 mg/mL). After 24 h, the medium was collected, and the levels of IL-1β, IL-6 and TNF-α were determined by Enzyme Linked Immuno Sorbent Assay—ELISA. The ELISA for IL-1β, IL-6 and TNF was realized with antibodies and standards from BD Pharmingen and follow the fabricant protocol.

### 3.9. Biotinylation of F2.0v

F2.0v was biotinylated as described in [13], using biotin-hydrazide (Pierce Chemical Co.; Rockford, IL, USA). Briefly, about 10 mg of F2.0v biotin-hydrazide (200 mmol) were dissolved in 20 mL HCl (0.1 M) pH 4.8. Then, 200 mmol of 1-(3′-dimetilaminopropil)-3-etilcarbodiimida (EDAC) (Sigma) was added. The pH of the reaction was kept at 4.8, with addition of HCl (0.01 M) for 60 min with stirring. The reaction was stopped by the addition of sodium acetate to a final concentration of 0.5 M, pH 4.8, and the solution stirred for an additional 60 min.

#### 3.9.1. Flow Cytometric Analysis

Cells (1 × 10^6^ in duplicate) obtained from peritoneal lavage were washed twice with RPMI (4 °C) medium and resuspended in 1 mL of phosphate buffer saline (PBS) (4 °C), containing saturating amounts of biotinylated F2.0v. After incubation for 1 h at 4 °C, cells were washed and suspended in a solution containing fluorescein isothiocyanate (FITC) (Molecular Probes; Eugene, OR, USA) conjugated with streptavidin. Again, after incubation for one hour, cells were washed and suspended in a solution containing anti-CD11a (Integrin αL Antibody—Santa Cruz Biotech., Dallas, TE, USA). After one hour of incubation, cells were washed tree times, as recommended by the manufacturer. Events (*n* = 40,000) were analyzed with a FACscan flow cytometry (Becton and Dickinson Immunocytometry System, San Jose, CA, USA).

Endothelial cells were harvested after a short exposure to 2 mM phosphate buffered saline-ethylene diamine tetra-acetic acid (EDTA-PBS). Cells (1 × 10^6^ per sample) were washed twice with F-12 medium and resuspended in 1 mL of PBS containing saturating amounts of biotinylated fucan. After incubation for 1 h at 4 °C, cells were washed and resuspended in a solution containing fluorescein isothiocyanate (FITC) (Molecular Probes; Eugene, OR) conjugated with streptavidin or WGA-FITC (5 mg/mL). Again, after incubation for 1 h, cells were washed and analyzed by flow cytometry (FACSCalibur, Becton and Dickinson, Sparks, MD, USA.

#### 3.9.2. Immunocytochemistry

Cells (1 × 10^6^ in duplicate) obtained from peritoneal lavage after washing were incubated with biotinylated F2.0v (10 mg/mL) or WGA-FITC (5 mg/mL) in the presence of 1% bovine serum albumin (BSA) (4 °C, 1 h). After washing several times, the cells were fixed (2% formaldehyde, 30 min, 22 °C) and washed several times. Biotinylated F2.0v was detected with streptavidin conjugated to Alexa Fluor 594 (5 mg/mL). After washing, the cells were incubated with DAPI (3 mM, 2 min), washed, mounted in Fluoromount-G (Electron Microscopy Sciences, Hatfield, PA, USA) and examined using a laser scanning confocal microscope (Zeiss LSM-510 NLO, Carl Zeiss, Weimar, Germany). In order to study the localization of different components, double-labeled experiments were performed.

The endothelial cells (1 × 10^5^) were placed on 12 mm-diameter glass cover slips in 24-well cluster plates (Nunc; Naperville, IL, USA). After 3 days in culture, the cells were washed three times with PBS (0.1 M pH 7.4), and the biotinylated fucan (10 μg/mL in PBS) was added to the cells before fixation (2% formaldehyde for 30 min). Fucan binding was revealed with Texas Red (Jackson ImmunoResearch, West Grove, PA, USA) conjugated streptavidin (5 μg/mL in PBS). The cells were then incubated with DAPI (1:2000) (Molecular Probes) for 2 min, washed five times in PBS, once in water, mounted in Fluoromount-G (E. M. Sciences; Ft. Washington, WA, USA) and examined with a confocal microscopy or fluorescence microscope.

#### 3.9.3. Acetic Acid-Induced Writhing Test

This was performed as described by Ribeiro *et al*. [32]. The animals were separated from the colony for a period of 12 h of fasting. Later, the animals were grouped (*n* = 5). The animals were treated prophylactically with F2.0v (0.25, 0.5, 1, 2, 4 and 20 mg/kg of animal) (i.v.). After 30 min, the pain induction was performed by administering 0.6% of acetic acid (i.p.). Quantitation was performed by painful cumulative count of the number of abdominal contractions occurring within 30 min after induction by stimulus of 0.6% of acetic acid. The positive control group received only 0.9% of saline i.v., and the negative control received dipyrone 20 mg/kg. 

#### 3.9.4. Hot Plate Test

This was performed according to Eddy and Leimbach [37]. The test was performed on hot plate (51.5 ± 1 °C). The animals passed under a screening, those showing sensitivity to temperature being discarded. The animals were treated prophylactically (30 min) with F2.0v at concentrations of 1.0 and 20.0 mg/kg of animal and immediately placed back on the hot plate for a verification response at time zero; the mice were placed back on the hot plate at intervals of 30, 60 and 90 min from the beginning of the experiment. To avoid tissue injury, the maximum exposure to the plate was 40 s. The test was conducted with groups of five animals. We used morphine 5 mg/kg of animal and saline 0.9% for positive and negative controls, respectively.

### 3.10. Statistical Analysis

All data were expressed as the mean ± standard deviation. Statistical analysis was performed by one-way ANOVA. Student-Newman-Keuls post-tests were carried out for multiple group comparison. In all cases, statistical significance was set at *p* < 0.05.

## 4. Conclusions

When taken together, the effects of F2.0v on nociception (the acetic acid-induced writhing) and inflammation shows significant peripheral antinociceptive and anti-inflammatory activity. We demonstrated for the first time that a heterofucan seaweed, *D. menstrualis* (F2.0v), showed antinociceptive and anti-inflammatory activities that are related to its ability to bind the cell surface of leukocytes, preventing the migration of these cells to the site of tissue injury. However, pharmacological studies are continuing in order to characterize the mechanism(s) responsible for the F2.0v antinociceptive and anti-inflammatory effects.
